# Potential contribution of enveloped virions to long-distance movement of plant rhabdoviruses

**DOI:** 10.3389/fmicb.2026.1994490

**Published:** 2026-10-19

**Authors:** Guowei Yang, Yinlu Deng, Qian Liu, Wenye Lin, Jingrui Wang, Li Xie, Zhenghe Li

**Affiliations:** 1 State Key Laboratory of Rice Biology and Breeding, Institute of Biotechnology, Zhejiang University, Hangzhou, China; 2 Ministry of Agriculture and Rural Affairs Key Laboratory of Molecular Biology of Crop Pathogens and Insect Pests, Zhejiang University, Hangzhou, China; 3 Zhejiang Key Laboratory of Biology and Ecological Regulation of Crop Pathogens and Insects, Zhejiang University, Hangzhou, China; 4 Guangzhou National Laboratory, Guangzhou, China; 5 Analysis Center of Agrobiology and Environmental Sciences, Zhejiang University, Hangzhou, China

**Keywords:** enveloped virion, phloem, rhabdovirus, sieve element, systemic movement, vessel element, xylem

## Abstract

Current models of plant virus movement are based largely on extensive studies of nonenveloped viruses, particularly those possessing positive-sense RNA genomes. In contrast, how enveloped, negative-sense RNA plant viruses access and transit through vascular tissues remains largely unexplored. Here, we aim to address this question using two rhabdovirus–host pathosystems: rice stripe mosaic virus (RSMV) infection of rice and tomato yellow mottle-associated virus (TYMaV) infection of *Nicotiana benthamiana*. Immunofluorescence staining of RSMV-infected rice tissues revealed that viral antigens were predominantly localized to vascular tissues, including both xylem and phloem. Transmission electron microscopy revealed that mature enveloped virions accumulated abundantly within xylem parenchyma cells. RSMV particles were also observed in two vascular conduits: xylem vessel lumens and sieve elements. Moreover, major viral structural proteins and morphologically intact virions were detected in xylem sap. Similarly, enveloped TYMaV virions were detected in both xylem vessel elements and phloem sieve elements of infected *N. benthamiana* plants. Furthermore, reverse genetics analyses demonstrated that a TYMaV matrix gene deletion mutant deficient in virion morphogenesis retained wild-type levels of cell-to-cell movement but was completely defective in long-distance movement. Together, these results indicate that intact rhabdovirus virions constitute an important mobile entity for vascular long-distance trafficking, with both phloem and xylem potentially contributing to systemic movement.

## 1 Introduction

Successful infection by plant viruses requires spread from initially infected cells to distal tissues and organs through two distinct phases: cell-to-cell movement through plasmodesmata (PD) and long-distance movement through the plant vascular system (Tilsner et al., 2014). After replication in initially infected cells, viral genomes, often packaged in a form of ribonucleoprotein complex (RNP), virion, or membranous viral replication complex (VRC), are delivered to PD. Virus-encoded movement proteins (MPs) increase the PD size exclusion limit and orchestrate intracellular trafficking of these infectious entities (Wu and Cheng, 2020; Kumar and Dasgupta, 2021; Alazem et al., 2026). This cycle repeats until the virus reaches the vascular bundles, where it enters conducting tubes for long-distance transport to distal organs. Genetic and cell biological evidence from numerous virus–host systems suggests that these two interconnected movement phases are mechanistically distinct and may employ different sets of viral and host proteins (Hipper et al., 2013; Tilsner et al., 2014).

The phloem has been considered as the principal route for viral long-distance movement. The phloem conduit consists of enucleated sieve elements (SE), which are metabolically supported by adjacent companion cells (CC) via specialized pore-plasmodesmal units (PPUs) (Oparka and Turgeon, 1999). To enter the phloem translocation stream for long-distance transport, plant viruses actively remodel PPUs to enable trafficking of infectious viral entities from CCs into SEs (Hipper et al., 2013; Kappagantu et al., 2020). Inside the phloem SEs, viruses undergo bidirectional long-distance translocation following the bulk flow of photoassimilates. Most viruses are thought to move as intact virions, because coat protein (CP) mutations that impair virion assembly typically abolish systemic infection (Brault et al., 2003; Zhang et al., 2013; Hipper et al., 2014). Indeed, virions of various positive-sense RNA viruses have been detected within SEs (Esau et al., 1967; Jensen, 1969; Halk and McGuire, 1973; Shepardson et al., 1980) or in phloem sap (Simón-Buela and García-Arenal, 1999; Requena et al., 2006). However, some viruses, such as tobacco rattle virus and cucumber green mottle mosaic virus, are assumed to move long distances as RNPs (Swanson et al., 2002; Savenkov et al., 2003; Hipper et al., 2013). Recently, membrane-associated VRCs of turnip mosaic virus (TuMV), a flexuous rod-shaped potyvirus, were identified in SEs and suggested to be the mobile form in the phloem (Wan et al., 2015; Wan and Laliberté, 2015; Movahed et al., 2017). Notably, virions of TuMV or related potyviruses have also been implicated in long-distance movement (Wang, 2021), suggesting that multiple forms of viral entities could be involved in systemic trafficking. Upon reaching distal sink tissues, viruses exit from the phloem tissues to reinitiate local replication and cell-to-cell movement. The molecular mechanisms governing viral phloem loading and unloading remain poorly characterized, with current evidence implying distinct regulatory pathways for these two processes (Kappagantu et al., 2020). For example, intact virions of turnip crinkle virus and tomato bushy stunt virus are dispensable for phloem entry but are required for phloem egress (Cao et al., 2010; Qu and Morris, 2002). Studies with phloem-restricted viruses indicate that phloem entry and exit are also shaped by active host defense pathways and viral counter-defense measures (Fagoaga et al., 2011; Gómez-Muñoz et al., 2017; Folimonova and Tilsner, 2018; Mei et al., 2026).

Apart from the phloem, the xylem has also been recognized as an alternative long-distance conduit (Sun et al., 2022). The xylem consists of two types of tracheary elements, vessel members and tracheids, which form hollow, dead conduits for unidirectional water and mineral transport from roots to aerial tissues. During tracheary element differentiation, living precursor cells undergo secondary wall thickening, lignification, and programmed cell death (PCD), losing all cytoplasmic contents (Schuetz et al., 2013; Růžička et al., 2015). To date, at least 39 plant viruses spanning diverse families, including potyviruses, closteroviruses, tobamoviruses, bromoviruses, comoviruses, geminiviruses, and a rhabdovirus, have been detected in xylem-associated cells, xylem sap, or guttation fluid (Sun et al., 2022 and references therein). Viral components detected in xylem tissues or fluids include CPs, inclusion bodies, intact virions, and vesicular VRCs (Opalka et al., 1998; French and Elder, 1999; Wan et al., 2015; Sun et al., 2022). In some cases, virus particles or viral antigens have been detected in both immature and mature xylem vessels (Chambers and Francki, 1966; Wan et al., 2015; Sun et al., 2022), supporting the notion that the xylem may function not only as a passive conduit for virions but also as a transient replication or assembly site during the course of xylem differentiation. Stem-girdling or steam treatment experiments that selectively destroy phloem while preserving xylem integrity have further confirmed that systemic infection can be achieved through xylem alone (Urban et al., 1989; Wan et al., 2015; Mochizuki et al., 2016).

Plant rhabdoviruses, are negative-sense, single-stranded RNA viruses comprising at least 269 species across 12 genera within the family *Rhabdoviridae* (Walker et al., 2026). Most plant rhabdoviruses possess bullet-shaped or bacilliform virions enveloped by a lipid bilayer and are transmitted by sap-feeding hemipteran insects in a persistent-propagative manner (Whitfield et al., 2018). Their genomes encode five conserved structural proteins, i.e., nucleoprotein (N), phosphoprotein (P), matrix protein (M), glycoprotein (G), and large polymerase (L), together with a non-structural protein MP (Jackson et al., 2005; Walker et al., 2011). During replication, the viral RNA is encapsidated by N in association with P and L to form nucleocapsids (NCs), which constitute the minimal infectious units for cell-to-cell movement (Wang et al., 2015; Jackson and Li, 2016). The rhabdovirus MPs, classified within the 30 K superfamily, interact specifically with their cognate N and P proteins to redirect NCs for intracellular and intercellular movement (Zhou et al., 2019a). During virion morphogenesis, the M protein condenses NCs into tightly coiled M–RNP complexes and drives budding through G-enriched host endomembrane to produce enveloped particles (Jayakar et al., 2004; Jackson et al., 2005; Sun et al., 2018). Based on their distinct replication sites, plant rhabdoviruses are conveniently categorized into nucleorhabdoviruses (nuclear replication) and cytorhabdoviruses (cytoplasmic replication), with mature virions accumulating within the luminal space of the nuclear envelope or endoplasmic reticulum (ER), respectively (Jackson et al., 2005). While NCs of plant rhabdoviruses are capable of cell-to-cell movement, the functional viral unit involved in vascular entry and systemic long-distance trafficking remains undefined.

In this study, we investigated the vascular trafficking mechanisms of two enveloped cytorhabdoviruses: rice stripe mosaic virus (RSMV) infecting rice and tomato yellow mottle-associated virus (TYMaV) infecting *Nicotiana benthamiana*. Taxonomically, RSMV belongs to the genus *Betacytorhabdovirus* (Yang et al., 2017) and TYMaV is classified within the genus *Alphacytorhabdovirus* (Xu et al., 2017). Combining immunofluorescence staining and transmission electron microscopy (TEM) assays, we detected enveloped virions of both viruses in xylem vessels and phloem sieve elements of systemically infected plants. Reverse genetics analyses revealed that a TYMaV M deletion mutant, which is incapable of completing virion morphogenesis, exhibited specific defects in systemic long-distance movement but retained intact local cell-to-cell propagation capacity. Collectively, these findings support the view that mature enveloped virions represent an important mobile entity in the vascular long-distance movement of plant rhabdoviruses.

## 2 Materials and methods

### 2.1 Plant and virus materials

Rice (*Oryza sativa* cv. ZH11) seedlings were grown in insect-proof growth chambers under conditions of 27–30 °C, a 16 h light/8 h dark photoperiod, and 60% relative humidity. *Nicotiana benthamiana* plants were grown at 25 °C under a 16 h light/8 h dark photoperiod. RSMV was maintained in rice plants through serial transmission by the insect vector *Recilia dorsalis*. The infectious clone of TYMaV with an inserted green fluorescence gene (pTYMaV-GFP) was previously described (Zhou et al., 2019a; Liang et al., 2023).

### 2.2 Plasmid construction

To generate an M-deficient TYMaV infectious clone, pTYMaV-GFP was digested with *Ahd*I and *Age*I to obtain the linearized vector backbone. Two fragments flanking the *M* coding region were amplified from pTYMaV-GFP using the primer pairs NF (5′-gtcgcttggaatgatgaggacg-3′) with RFPNR (5′-caccatataacgtgttattcacctgctg-3′), and RFPGF (5′-gtacaagtaagcgctgtcatagtgagatctag-3′) with LR (5′-aggtgtttcgcaagaaccg-3′). The mCherry coding sequence was amplified from a plasmid using RFPF (5′-taacacgttatatggtgagcaagggcgag-3′) and RFPR (5′-acagcgcttacttgtacagctcgtccatgc-3′). The linearized vector backbone and the three PCR fragments were assembled by In-Fusion cloning to generate the TYMaV-ΔM:RFP infectious clone (pTYMaV-GFP-ΔM:RFP).

### 2.3 Agrobacterium-mediated virus inoculation

Agrobacterium-mediated inoculation was performed as described previously (Zhou et al., 2019a), with minor modifications. Briefly, *Agrobacterium tumefaciens* strain EHA105 cultures carrying the full-length TYMaV cDNA clone, pGD-N, pGD-P, pCB301-L, and the silencing suppressors pGD-P19, pGD-HcPro, and pGD-γb were mixed and adjusted to final OD_600_ values of 0.24, 0.08, 0.08, 0.24, 0.08, 0.08, and 0.08, respectively. The mixed suspensions were infiltrated into leaves of approximately four-week-old *N. benthamiana* plants. For cell-to-cell movement assays, the above Agrobacterium cell mixtures were diluted 150-fold before infiltration. Inoculated plants were maintained under greenhouse conditions and used for subsequent analyses at the indicated time points.

### 2.4 Collection of xylem sap

Xylem sap was isolated from rice roots and stems using a modified protocol as previously described (Wan et al., 2015). Briefly, approximately 70 g of root and stem tissues were separately harvested and cut into 5-cm segments. The segments were rinsed three times with deionized water and gently blotted dry with paper towel to eliminate contaminants derived from cut surfaces. The processed tissues were transferred to centrifuge tubes and fully immersed in pre-prepared extraction buffer consisting of 0.1 M Tris–HCl (pH 7.6) and 0.2 M KCl, supplemented with fresh protease inhibitor cocktail (Roche, 4,693,116,001) immediately before use. Samples were subjected to vacuum treatment at 75 kPa for 2 min, followed by gradual pressure release. After removing excess buffer from the tissues on paper towel, the inner sample tubes were nested in collection tubes and centrifuged at 1,000 × g for 20 min at 4 °C. The harvested xylem sap was partially concentrated using Microcon YM-3 centrifugal filter units (Millipore), with a portion of the unconcentrated sap reserved for immunoblotting assays. Viral particles were pelleted from pooled root and stem xylem sap via ultracentrifugation at 93,000 × g for 30 min at 4 °C, and the resulting pellet was resuspended in 700 μL of maintenance buffer (0.1 M Tris–HCl, pH 7.5, 10 mM magnesium acetate, and 1 mM MnCl₂).

The activity of glucose-6-phosphate dehydrogenase (G6PDH), a specific cytoplasmic marker, was quantified using a commercial G6PDH Activity Assay Kit (Solarbio, BC0260) in accordance with the manufacturer’s protocols. Total protein extracts from intact rice tissues prepared with the identical extraction buffer were used as positive controls. Enzymatic reactions were incubated at 37 °C, and the absorbance at 340 nm was measured at 30-s intervals for 8 min to calculate the relative G6PDH activity in all samples.

### 2.5 Virus particle purification

RSMV particles were purified from infected rice tissues by sucrose density gradient centrifugation as described previously (Jackson and Christie, 1977; Sun et al., 2018), with minor modifications. Briefly, 30–50 g of infected plant tissues was homogenized in approximately two volumes of extraction buffer (0.1 M Tris–HCl, pH 8.4, 10 mM magnesium acetate, 1 mM MnCl₂, and 40 mM Na_2_SO_3_) and filtered through two layers of cheesecloth. After clarification by centrifugation at 6,000 g for 10 min at 4 °C, the supernatant was layered onto a discontinuous 30%/60% (w/v) sucrose gradient and centrifuged at 80,000 g for 90 min at 4 °C in a Type 70 Ti rotor (Beckman Coulter). The fraction at the interface between the 30 and 60% sucrose layers was collected, diluted with maintenance buffer, and further clarified by filtration through diatomaceous earth (Celite). Virus particles were subsequently pelleted by centrifugation at 80,000 g for 30 min at 4 °C in a Type 70 Ti rotor and gently resuspended in maintenance buffer. The suspension was layered onto a 5–30% (w/v) continuous sucrose gradient and centrifuged at 54,000 g for 50 min at 4 °C in an SW 41 Ti rotor. Fractions containing virus particles were pooled and subjected to a final centrifugation at 80,000 g for 30 min at 4 °C. The resulting virus pellet was gently resuspended in maintenance buffer and analyzed by SDS–PAGE followed by Coomassie Brilliant Blue staining to visualize viral structural proteins.

### 2.6 Immunoblot analysis

Plant tissues were frozen in liquid nitrogen and ground into a fine powder. Approximately 0.1 g of tissue powder was homogenized in 200 μL of total protein extraction buffer containing 1 M Tris–HCl (pH 6.8), 4.5% SDS, and 8 M urea. After centrifugation at 12,000 × g for 10 min, the supernatant was collected as the total protein extract.

Protein samples were separated by SDS–PAGE and transferred onto PVDF membranes by wet transfer at 100 V for 75 min. Membranes were blocked with 5% non-fat milk in PBST buffer (3.2 mM Na_2_HPO_4_, 0.5 mM KH_2_PO_4_, 1.3 mM KCl, 135 mM NaCl, 0.05% Tween 20, pH 7.4) for 1 h and incubated with primary antibodies against purified RSMV virions, RSMV N protein (Guo et al., 2020), or GFP (Abcam, ab32146) at 4 °C overnight. After three washes with PBST for 10 min each, membranes were incubated with horseradish peroxidase-conjugated secondary antibodies, followed by three additional washes with PBST. Immunoreactive signals were detected using an enhanced chemiluminescence (Fdbio, FD8020) substrate and visualized using an ImageQuant LAS 4000 mini imaging system (GE Healthcare). After signal acquisition, PVDF membranes were stained with Ponceau S to verify protein transfer and loading consistency.

### 2.7 Cryosectioning and immunofluorescence staining

Leaf and stem tissues were collected from healthy and RSMV-infected rice plants and immediately immersed in 4% paraformaldehyde (PFA) prepared in phosphate-buffered saline (PBS). Samples were vacuum infiltrated to facilitate penetration of the fixative and then fixed overnight at 4 °C. Following fixation, the tissues were sequentially infiltrated with 10%, 20%, and 30% sucrose solutions prepared in PBS containing 0.4% Triton X-100, with each step performed for 1 h at 4 °C. The tissues were then incubated in a 1:1 mixture of 50% sucrose solution and optimal cutting temperature (OCT) compound for 30 min–1 h at room temperature. After infiltration, the tissues were embedded in OCT compound, snap-frozen in liquid nitrogen, and maintained at −80 °C for 30 min before cryosectioning.

The embedded tissues were sectioned at a thickness of approximately 15 μm using a cryostat and collected onto microscope slides. Sections were gently washed three times with PBS for 5 min each and blocked with 5% bovine serum albumin (BSA) for 30 min at room temperature. The sections were then incubated with an RSMV N protein-specific mouse monoclonal antibody (1:5,000; Guo et al., 2020) overnight at 4 °C. After three washes with PBS, the sections were incubated with Alexa Fluor 555-conjugated goat anti-mouse IgG (H + L) secondary antibody (Invitrogen, A-21422) for 1 h at room temperature in the dark, followed by three additional washes with PBS.

### 2.8 Confocal and fluorescence microscopy

The immuno-stained sections were mounted in a small volume of PBS, covered with coverslips, and immediately examined using an LSM 780 confocal microscope (Zeiss, Germany). Alexa Fluor 555 was excited with a 561-nm laser, and fluorescence emission was collected at 570–620 nm.

Leaf tissues infected with recombinant viruses were examined with a SteREO Lumar V12 imaging system (Zeiss, Germany). GFP and RFP emissions were captured using filter sets 38 (470/40 nm excitation, 525/50 nm collection) and 31 (565/30 nm excitation, 620/60 nm collection), respectively. Image analysis was conducted using ZEN 2.3 (blue edition) software.

### 2.9 Transmission electron microscopy (TEM)

For ultrastructural observation of plant tissues, samples were collected from four independently infected plants for each virus–host system. Freshly excised tissue pieces (approximately 1 × 1 × 3 mm) were immediately immersed in 2.5% glutaraldehyde and vacuum-infiltrated for 15–20 min to facilitate fixative penetration, followed by fixation overnight at 4 °C. Samples were washed three times with 0.1 M phosphate buffer (pH 7.0) for 15 min each and postfixed in 1% osmium tetroxide for 1.5 h. After three additional washes with 0.1 M phosphate buffer (pH 7.0), samples were dehydrated sequentially in 30%, 50%, 70%, and 80% ethanol for 15 min each, followed by 90 and 95% acetone for 15 min each and two changes of 100% acetone for 20 min each. Dehydrated samples were infiltrated sequentially with acetone/Spurr resin mixtures at ratios of 1:1 (v/v) for 1 h and 1:3 (v/v) for 3 h, and then transferred to 100% Spurr resin for overnight infiltration at room temperature. Samples were embedded in fresh Spurr resin and polymerized at 70 °C for 48 h. Ultrathin sections were prepared from seven resin-embedded blocks from RSMV-infected rice and nine from TYMaV-infected *N. benthamiana* using a Leica EM UC7 ultramicrotome (Leica Microsystems, Germany), double-stained with uranyl acetate and lead citrate, and examined using a Hitachi H-7650 or HT7820 transmission electron microscope (Hitachi High-Tech, Japan).

For negative-stain TEM, liquid samples were applied to film-coated copper grids and allowed to adsorb for 3 min. Excess liquid was removed with filter paper, and the grids were fixed with 2.5% glutaraldehyde for 30 s and rinsed with double-distilled water. The grids were then negatively stained with 2% phosphotungstic acid (PTA; pH 6.7) for 3 min. Excess stain was removed with filter paper, and the grids were air-dried before examination with a Hitachi H-7650 transmission electron microscope (Hitachi High-Tech, Japan).

### 2.10 Immuno-gold labeling assay

For ultrastructural immuno-gold labelling of viral structures, fresh plant tissues were cut into small blocks (1 × 1 × 3 mm) and immediately fixed in a mixed solution containing 4% paraformaldehyde and 0.2% glutaraldehyde. Vacuum infiltration was applied for 15–20 min to promote full penetration of fixatives, followed by sustained fixation at 4 °C for 4 h. After thorough rinsing three times with 0.1 M phosphate buffer (pH 7.0), tissue dehydration was performed via a graded ethanol series. Samples were sequentially equilibrated in 30 and 50% ethanol (30 min each at 4 °C), followed by serial incubation in 70%, 90%, and three rounds of 100% ethanol (1 h per step at −20 °C). For resin embedding, dehydrated samples were gradually infiltrated with ethanol/K4M resin mixtures at volumetric ratios of 1:1 for 2 h and 1:3 for 4 h, and finally immersed in pure K4M resin overnight at −20 °C. Fully infiltrated samples were embedded in fresh K4M resin and polymerized under 360 nm ultraviolet irradiation, with sequential treatment at −20 °C for 72 h and at room temperature for 48 h. Ultrathin sections were generated using a Leica EM UC7 ultramicrotome (Leica Microsystems, Germany). For immunolabeling, tissue sections were pre-blocked with commercial blocking buffer (EMS, Hatfield, PA) at 37 °C for 30 min to eliminate non-specific binding. Blocked sections were subsequently probed with primary antibody (1:50 dilution in blocking buffer) raised against TYMaV P protein (Liang et al., 2023) overnight at 4 °C. After three rounds of ultrapure water washing (5 min per wash), sections were incubated with a 1:100-diluted IgG-gold conjugate as the secondary antibody for 2 h at 37 °C, followed by additional triple rinses with ultrapure water. Sections were double-stained with uranyl acetate and lead citrate for ultrastructural contrast. Final labeling signals and subcellular ultrastructures were visualized and imaged using a Hitachi H-7650 transmission electron microscope. For negative control validation, parallel sections were incubated with non-immune serum in place of the primary antibody under identical experimental conditions.

## 3 Results

### 3.1 Localization of RSMV in rice vascular tissues

To characterize the spatial distribution of RSMV in systemically infected tissues, healthy and RSMV-infected rice plants were collected and processed for immunofluorescence analysis (Figure 1A). Transverse cryosections of leaf and stem tissues were stained with an RSMV N protein-specific antibody (Guo et al., 2020) and examined by confocal microscopy. The vascular architecture of RSMV-infected rice tissues was largely comparable to that of healthy controls, containing xylem vessels with wide lumens, phloem cell clusters, and surrounding bundle sheath cells (Figures 1B,C). No obvious N protein signal was detected in sections from healthy plants, whereas strong red fluorescence was observed in RSMV-infected samples, confirming the specificity of the immunofluorescence assay.

**Figure 1:**
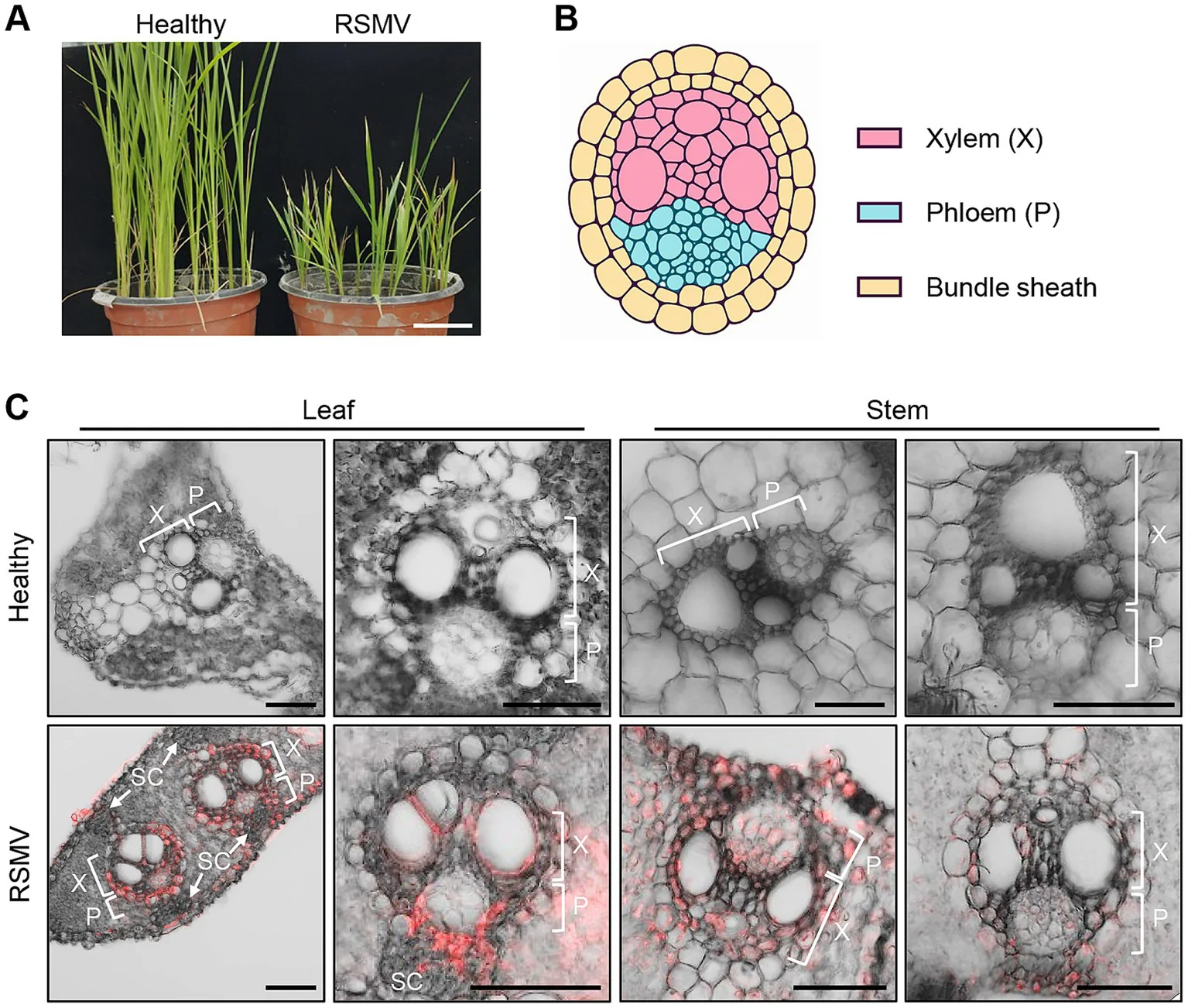
Immunofluorescence microscopy analysis of the distribution of RSMV N antigen in rice tissues. **(A)** Phenotypes of healthy and RSMV-infected rice plants. Scale bar, 3 cm. **(B)** Simplified schematic showing a transverse section of a rice vascular bundle containing the xylem (pink), phloem (blue), and bundle sheath (yellow). **(C)** Immunofluorescence staining of RSMV N protein in transverse cryosections of stems and leaves from healthy and RSMV-infected rice plants. X, xylem; P, phloem; Sc, sclerenchyma. Scale bars, 50 μm.

In sections of infected leaves, RSMV N protein signals were detected primarily in the vasculature, including xylem and phloem-associated regions and surrounding parenchyma and bundle sheath cells. Antigen staining was also observed in sclerenchyma and, sporadically, in mesophyll cells (Figure 1C). Within vascular bundles, positive staining was frequently detected in association with large xylem vessels. Comparable RSMV distribution patterns were observed in stem sections of infected plants. Collectively, these results demonstrate that RSMV N proteins or NCs are broadly distributed throughout the vascular tissues of rice during systemic infection. However, the immunofluorescence assays do not resolve the structural form of the virus at these locations.

### 3.2 Ultrastructural localization of RSMV virions in xylem tissues

To further resolve RSMV localization at the cellular level in rice xylem tissues, ultrathin sections of RSMV-infected rice stems were examined by TEM. Representative images revealed that mature enveloped RSMV virions, approximately 50–60 nm in diameter, occurred in clusters enclosed in vesicle-like membranes; non-enveloped particles (cores), approximately 20–25 nm in diameter, were also occasionally observed (Supplementary Figure S1). The morphology of these particles was consistent with that described previously (Yang et al., 2017; Zhao et al., 2019; Wu et al., 2026a).

In transverse sections of infected rice stems, virus particles were particularly abundant in xylem parenchyma cells surrounding mature vessel elements (VEs) in both the protoxylem and metaxylem (Figure 2A). Higher-magnification imaging revealed that most of the observed particles were mature enveloped virions arranged in orderly arrays within an enclosing membrane, densely accumulating and occupying a substantial proportion of the cytoplasmic space.

**Figure 2:**
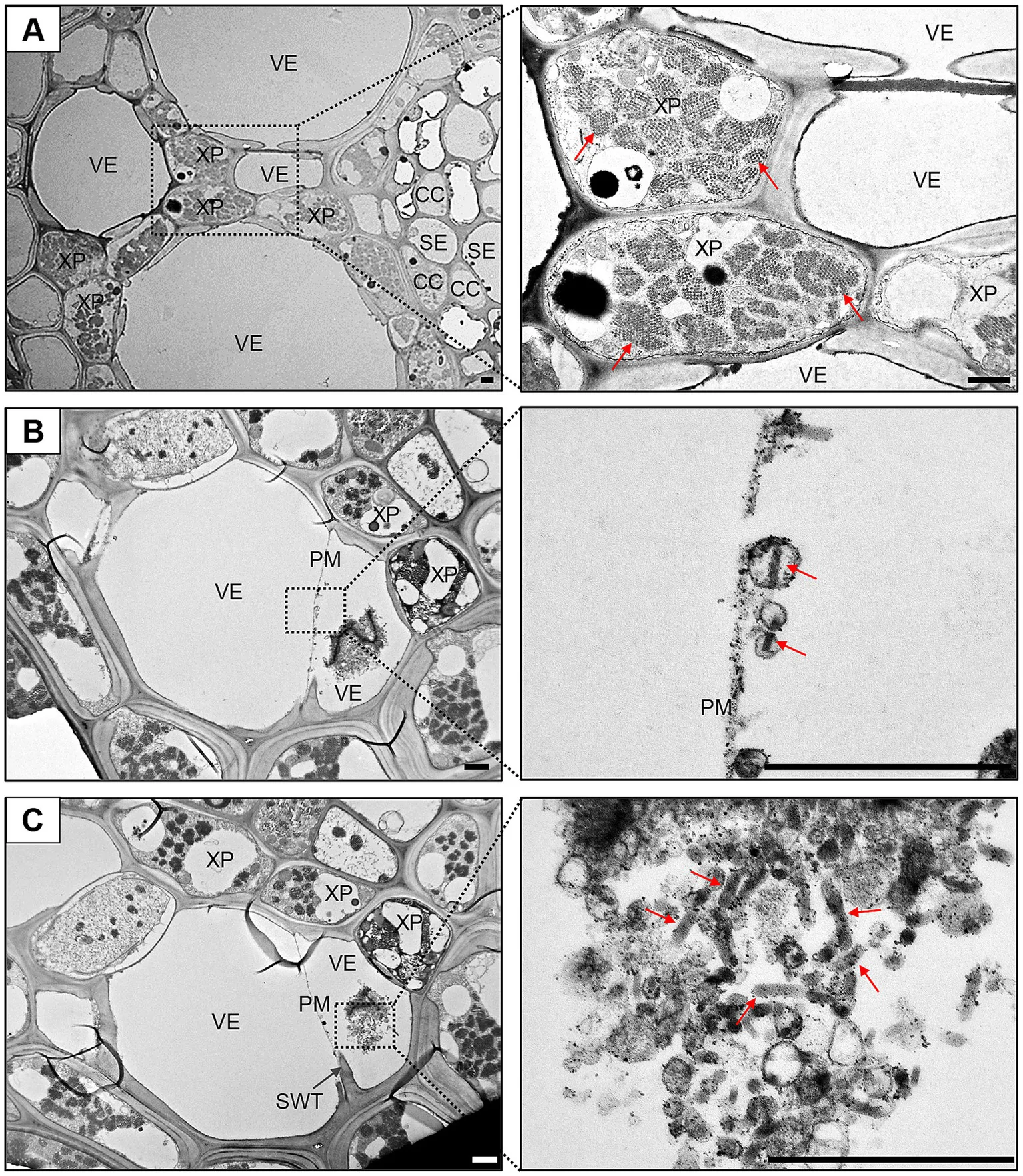
TEM observation of RSMV particles in rice xylem tissues. **(A)** RSMV virions in xylem parenchyma cells. **(B,C)** Enveloped RSMV virions in vessel elements, enclosed within vesicles in **(B)** and without evident surrounding vesicles in **(C)**. Boxed regions are shown at higher magnification, and red arrows indicate virus particles. CC, companion cell; SE, sieve element; SWT, secondary cell wall thickening; XP, xylem parenchyma cell; VE, vessel element; PM, pit membrane. Scale bars, 1 μm.

Mature tracheary elements undergo complete protoplast clearance and function as hollow conduits for water transport (Schuetz et al., 2013), which renders the fixation and detection of material within their large lumens technically challenging. Nevertheless, bacilliform-like particles with or without a enclosing membrane were observed within the lumen of mature VEs (Figures 2B,C; Supplementary Figure S2). These particles measured approximately 48–55 nm in diameter, consistent with the morphology and dimensions of RSMV enveloped virions. Such particles were detected in 5 out of 46 VE-containing TEM fields.

To substantiate the ultrastructural observations, xylem sap was collected from the stem and root sectors of RSMV-infected rice plants and analyzed for the presence of viral structural components and particles. Protein gel blotting with polyclonal antisera raised against purified RSMV virions detected four major protein bands in stem samples, whose migration patterns and relative abundances matched those of the N, P, M, and G structural proteins in purified virion preparations (Figure 3A). Root samples contained distinct G and M protein signals, whereas the N and P proteins appeared as smears. Importantly, negative-stain TEM of concentrated xylem sap further revealed bacilliform particles approximately 65 nm in diameter, exhibiting morphology characteristic of plant rhabdovirus virions (Figure 3B). The slightly larger apparent diameter relative to that observed in resin-embedded tissue sections likely reflects differences in sample preparation and staining (Kurz et al., 1986; Mast and Demeestere, 2009). A G6PDH activity assay excluded any significant cytoplasmic contamination in the stem and root xylem sap samples (Supplementary Figure S3).

**Figure 3:**
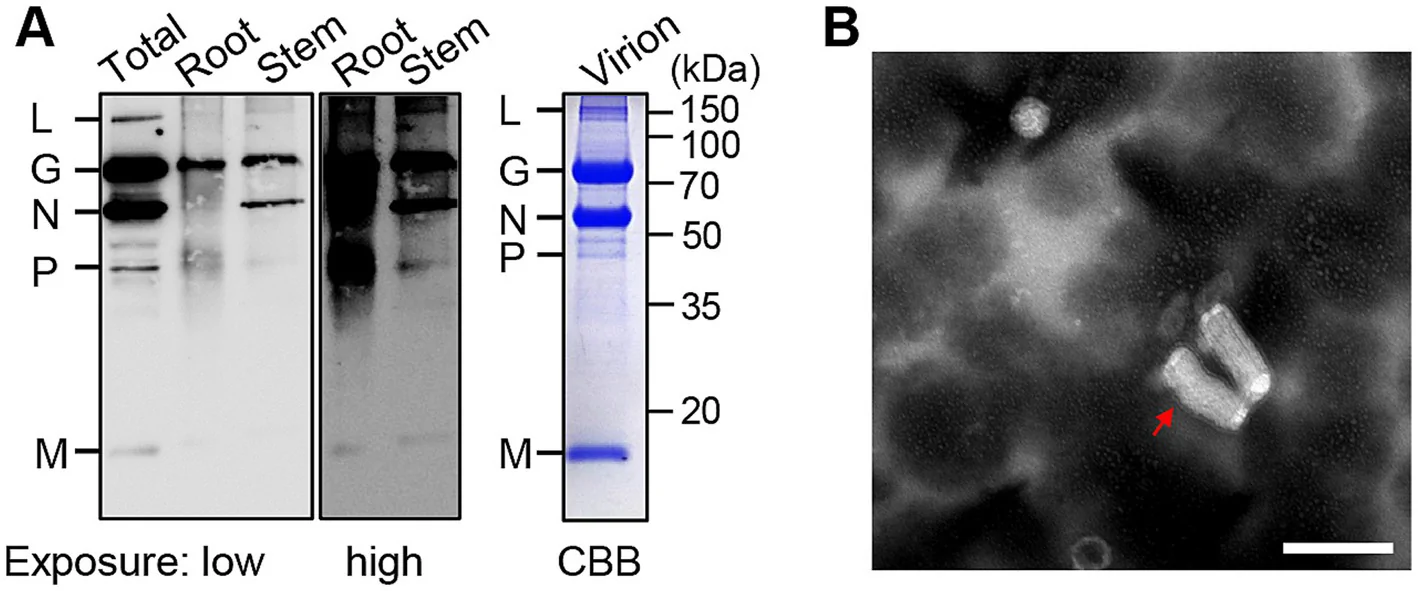
Detection of RSMV particles in rice xylem sap. **(A)** Immunoblot analysis of RSMV structural proteins in xylem fluid collected from infected rice plants. A short exposure (left panel) and long exposure (middle panel) of the blot were shown, and Coomassie Brilliant Blue (CBB) staining of purified RSMV virions is represented as a reference for the migration positions of the viral structural proteins (right panel). Positions of viral structural proteins and molecular mass markers are indicated. **(B)** Negative-stain TEM of concentrated xylem sap showing bacilliform RSMV particles. Scale bar, 200 nm.

Like xylem VEs, sclerenchyma cells also undergo PCD, deposit thickened secondary cell walls, and become dead at maturity (Beers, 1997; Matsuo and Hoshikawa, 1993). Given the positive N protein staining in hypodermal sclerenchyma tissues (Figure 1C), we next examined the viral structures present in these cells by TEM. In transverse sections of infected rice leaves, membrane-enclosed mature virions were detected in sclerenchyma cells retaining little cytoplasmic content (Supplementary Figure S4). Together, these observations suggest that mature RSMV particles can persist during advanced stages of cellular autolysis.

### 3.3 Ultrastructural localization of RSMV virions in phloem sieve element–companion cell complexes

Given the detection of RSMV N protein signals in phloem tissues by immunofluorescence staining (Figure 1C), we further examined the ultrastructural localization of RSMV in phloem cells of infected rice stems. Mature SEs exhibited relatively sparse cytoplasmic contents and a limited complement of organelles, a consequence of selective autolysis during differentiation (Heo et al., 2017; Knoblauch and Oparka, 2012). SE-specific plastids (SEPs) and PPUs at SE–CC interfaces serve as diagnostic features for identifying SEs and their adjacent CCs (Figure 4).

**Figure 4:**
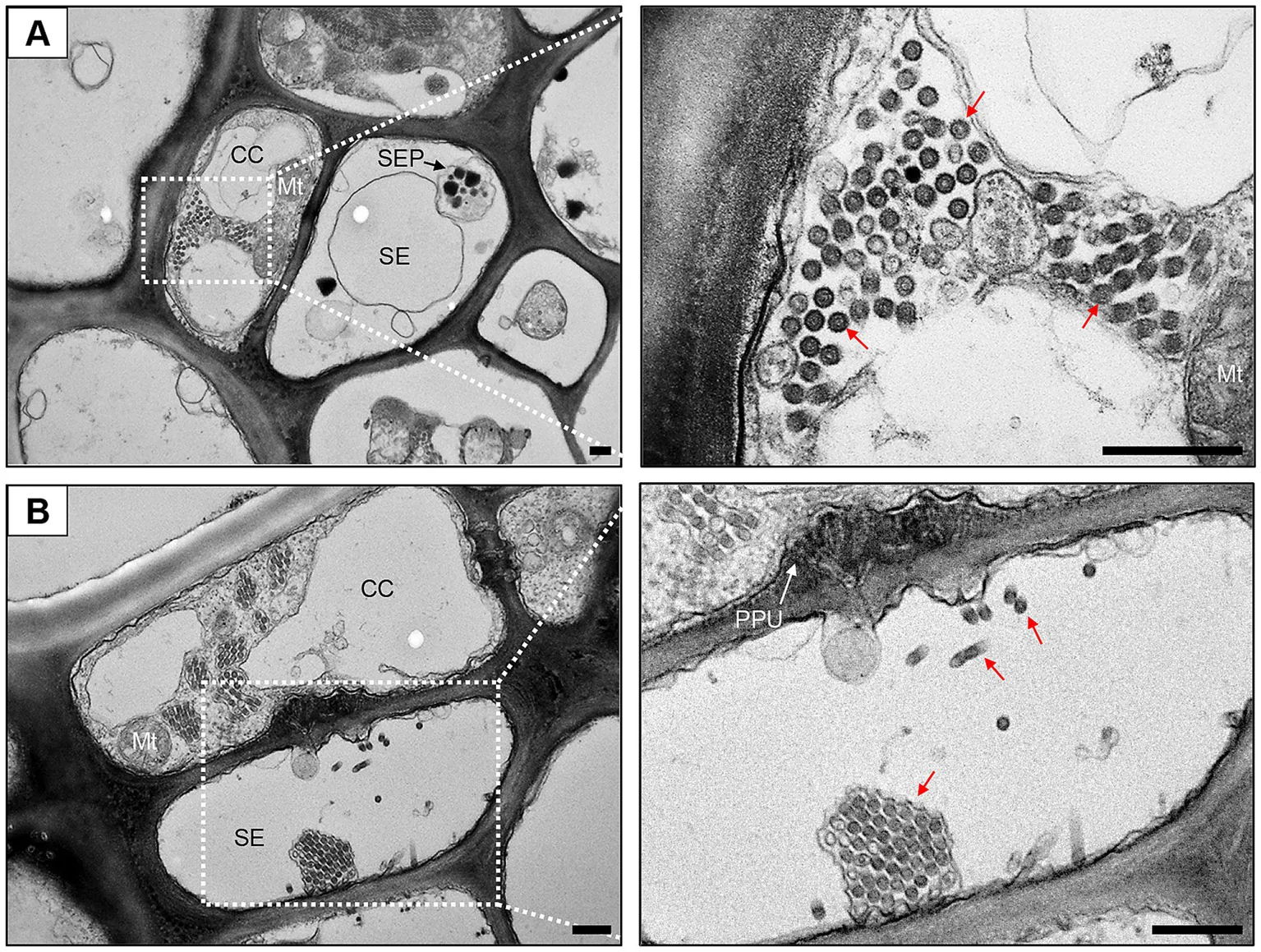
Ultrastructure of the phloem tissues in rice stem infected with RSMV. Dashed boxes are enlarged at right to show enveloped virions present in a companion cell **(A)** and a sieve element **(B)**. Red arrows and black arrows indicate enveloped virions with or without a surrounding membrane. CC, companion cells; PPU, pore-plasmodesma unit; SE, sieve element; SEP, sieve element-specific plastid. Scale bars, 500 nm.

Representative SE–CC complexes containing virus particles were identified among the examined sections (Figure 4; Supplementary Figure S5). At higher magnification, bacilliform particles morphologically consistent with mature enveloped RSMV virions were observed within both CCs (Figure 4A) and SEs (Figure 4B). In SEs, most virions occurred in clusters enclosed by a vesicle-like membrane, whereas a small number of individual enveloped virions lacking a visible surrounding membrane were also observed. Among 62 SE-containing fields examined, RSMV virions were identified in 8 independent fields. These observations demonstrate that mature RSMV particles are also present within phloem SE–CC complexes during systemic infection.

### 3.4 Presence of TYMaV virions in xylem vessel elements and phloem sieve elements

To determine whether the observed vascular localization patterns of RSMV virions also occur in another rhabdovirus–host system, we examined the ultrastructure in TYMaV-infected *N. benthamiana* (Figure 5A). TEM examination of systemically infected mesophyll tissues revealed cytoplasmic vesicles containing mature bacilliform virions, approximately 50–60 nm in width; naked NC cores that had not yet undergone envelopment, measuring 20–30 nm in diameter, were also observed in orderly arrays (Supplementary Figure S6).

**Figure 5:**
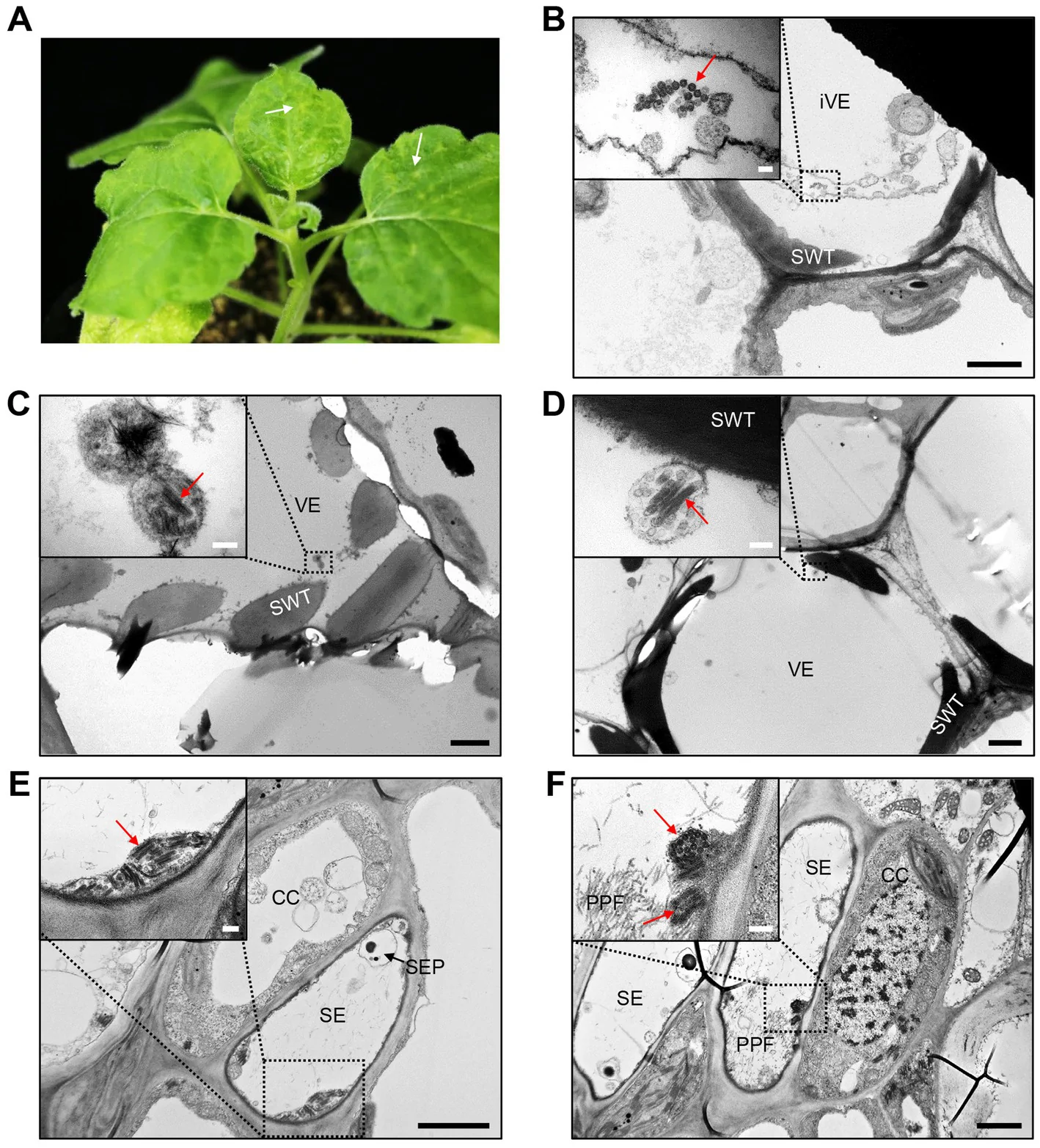
Detection of TYMaV virions in the vasculature of *Nicotiana benthamiana* leaf veins. **(A)** Symptoms of a TYMaV-infected *N. benthamiana* plant. White arrows indicate leaf mottling and wrinkling. **(B–D)** Ultrastructure of the xylem tissues in TYMaV-infected *N. benthamiana* leaves. Enveloped TYMaV particles were detected in a transverse section of an immature vessel element (iVE) **(B)** and in longitudinal **(C)** and transverse **(D)** sections of mature vessel elements (VEs). **(E–F)** Transverse section of sieve element (SE)–companion cell (CC) complexes showing enveloped TYMaV virions within SEs. Some virions in **(F)** are enclosed within vesicles. Dashed boxes indicate regions shown at higher magnification, and red arrows indicate virions. SWT, secondary cell wall thickening; SEP, sieve element-specific plastid; PPF, P-protein filaments. Black and white scale bars are 2 μm and 100 nm, respectively.

In ultrathin sections of infected leaf veins, enveloped virions were identified within immature vessel elements (iVEs) (Figure 5B). These cells exhibited patterned secondary cell wall thickening while retaining residual cytoplasmic contents, consistent with an intermediate stage of tracheary element differentiation (Schuetz et al., 2012; Groover et al., 1997). Furthermore, mature virions enclosed in clearly defined vesicle-like membrane structures were also observed within fully differentiated VEs in both longitudinal and transverse sections (Figures 5C,D; Supplementary Figure S7). These hollow cells displayed prominent patterned wall thickening and no apparent protoplasmic contents. In the phloem, enveloped TYMaV virions were detected within SEs, with or without an evident surrounding vesicle (Figures 5E,F; Supplementary Figure S8). Across the TEM observations, 50 fields containing iVEs and/or mature VEs and approximately 58 SE-containing fields were examined, and enveloped virions were observed in at least 3 immature VEs, 7 mature VEs, and 6 independent SE-containing fields. Collectively, these observations demonstrate that mature enveloped TYMaV virions are present in both immature and fully differentiated VEs, as well as in SEs, during systemic infection.

### 3.5 Specific defect in systemic infection of a TYMaV M deletion mutant unable to form virions

We next investigated the relationship between mature virion formation and TYMaV systemic infection. The parental TYMaV infectious clone carries a green fluorescent protein (GFP) reporter inserted between the N and P open reading frames (ORFs) (Liang et al., 2023), enabling direct visualization and tracking of infected cells. Given the established role of the rhabdovirus M protein in virion assembly and budding (Jayakar et al., 2004; Ma and Li, 2020), we generated TYMaV-ΔM:RFP, in which the M ORF was replaced by a red fluorescent protein (RFP) reporter while the GFP reporter was retained (Figure 6A).

**Figure 6:**
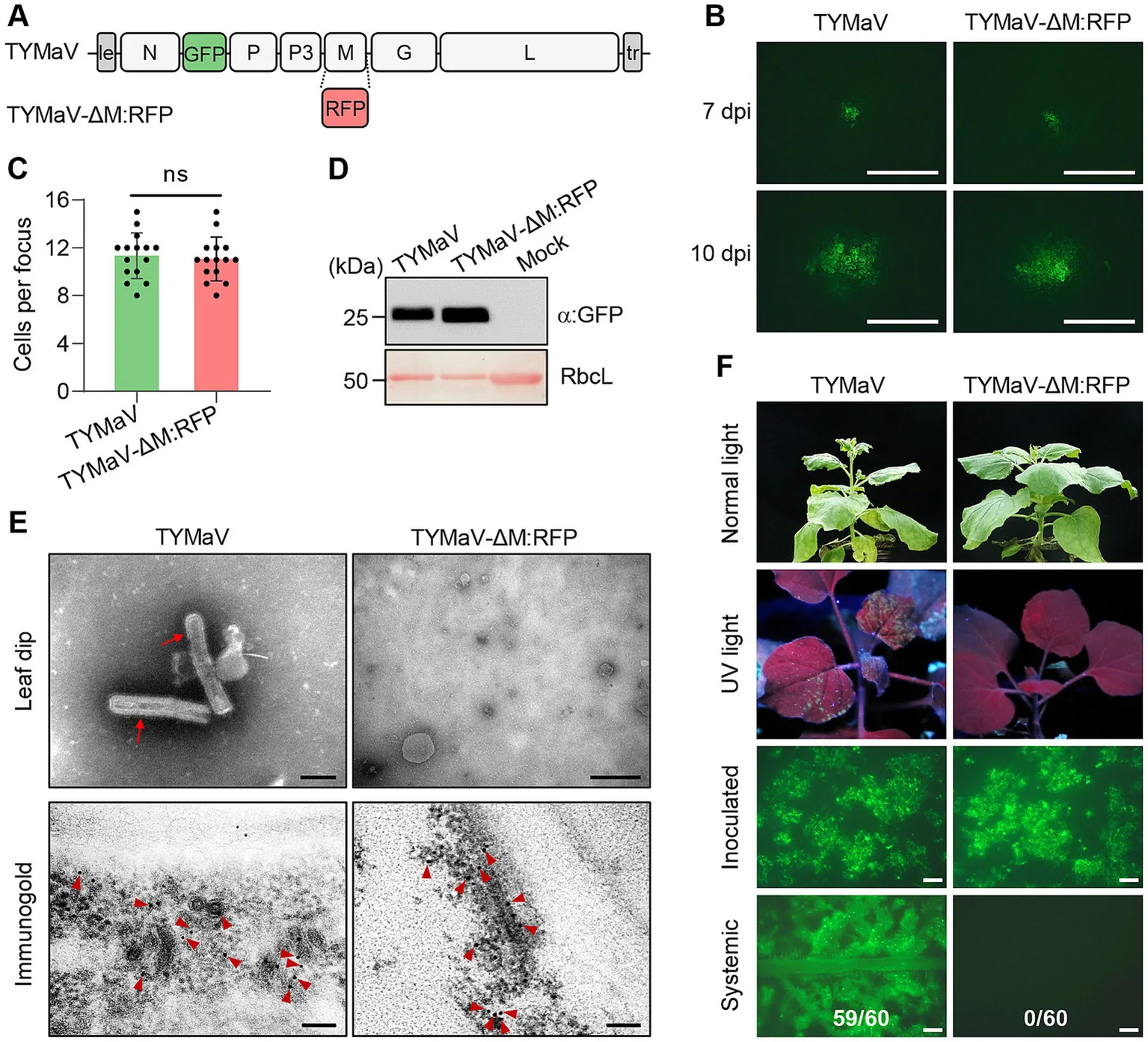
Reverse genetic analyses of the infection phenotypes of TYMaV-ΔM:RFP. **(A)** Schematic representation of the genome structures of TYMaV and TYMaV-ΔM:RFP containing a *GFP* gene inserted between the *N* and *P* genes. **(B–D)** Analyses of cell-to-cell movement of TYMaV and TYMaV-ΔM:RFP. **(B)** Agroinoculated leaves were monitored under a fluorescence microscope at 7 dpi and 10 dpi. **(C)** The number of cells per infection focus was quantified at 10 dpi. Statistical significance was assessed using a two-tailed unpaired Student’s *t*-test; ns, *p* > 0.05. **(D)** The accumulation of GFP protein in leaf tissues inoculated with TYMaV and TYMaV-ΔM:RFP was detected by immunoblotting at 14 dpi. RbcL staining is shown as a loading control. **(E)** Analyses of virion formation by leaf-dip negative-stain (top) and immunogold TEM (bottom) in the leaf tissues inoculated with TYMaV or TYMaV-ΔM:RFP. Immunogold labeling was performed using an antibody against the TYMaV P protein. Arrows indicate TYMaV virions in leaf-dip preparations, and arrowheads indicate gold particles. **(F)** Comparison of infection phenotypes of TYMaV or TYMaV-ΔM:RFP. Plant symptoms under normal light and handheld UV illumination were recorded at 17 and 12 dpi, respectively. Fluorescence stereomicroscopy of inoculated and upper systemic leaves was performed at 6 and 15 dpi, respectively. Numbers indicate the number of systemically infected plants per total inoculated plants. Scale bars are 500 μm **(B, F)** and 100 nm **(E)**.

To assess cell-to-cell movement, *N. benthamiana* leaves were inoculated with diluted agrobacterial cultures harboring the virus infectious cDNA clones. At approximately 6–7 days post inoculation (dpi), isolated GFP-positive single cells became visible in leaves inoculated with either TYMaV or TYMaV-ΔM:RFP, representing initial infection foci (Figure 6B). These foci subsequently expanded, reaching approximately 12 cells on average by 10 dpi for both the wild-type virus and the M deletion mutant (Figures 6B,C). Immunoblot analysis of inoculated leaves showed that GFP accumulated to slightly lower levels in TYMaV than in TYMaV-ΔM:RFP-infected tissues (Figure 6D), likely reflecting the ability of M to suppress viral RNA synthesis (Zhou et al., 2019b; Jiang et al., 2025). Nevertheless, these results indicate that deletion of M did not appreciably impair cell-to-cell movement.

To determine whether M deletion disrupted virion formation, inoculated leaf tissues were examined by negative-stain and immunogold TEM analyses. Negative-stain TEM of leaf dips readily detected mature bacilliform virions in TYMaV-infected leaves, whereas such particles were not detected in TYMaV-ΔM:RFP-infected leaves (Figure 6E). Immunogold TEM using TYMaV P protein-specific antibody conjugate further detected specific labeling signals associated with morphologically intact mature virions in TYMaV-inoculated leaves; in TYMaV-ΔM:RFP-inoculated cells, by contrast, gold labeling remained readily detectable but was distributed over viral material lacking recognizable virion morphology (Figure 6E). These complementary observations indicate that the M deletion mutant failed to assemble into detectable virions.

Under agroinoculation conditions permitting systemic infection, GFP fluorescence was readily detected in inoculated leaves at 6 dpi and had spread extensively by 15 dpi in leaves inoculated with either TYMaV or TYMaV-ΔM:RFP. From approximately 10 dpi onward, TYMaV-inoculated plants progressively developed systemic GFP fluorescence, followed by typical mottling and leaf distortion in upper leaves. In contrast, no systemic GFP fluorescence or obvious symptoms were detected in TYMaV-ΔM:RFP-inoculated plants throughout the observation period (Figure 6F). Systemic infection was detected in 59 of 60 TYMaV-inoculated plants by 21 dpi, whereas none of the 60 TYMaV-ΔM:RFP-inoculated plants became systemically infected. Thus, deletion of TYMaV *M* gene prevented detectable virion formation and abolished systemic infection without appreciably affecting local cell-to-cell movement.

## 4 Discussion

Unlike mammalian rhabdoviruses, which typically bud from the plasma membrane to release mature virions into the extracellular space for infection of neighboring cells, plant-infecting rhabdoviruses bud into endomembrane compartments and assemble virions that accumulate within the nuclear envelope or ER (Jackson et al., 2005). In plants, the nuclear envelope and ER form a continuous membrane network with desmotubules, which are tubular ER-derived structures that traverse the central axis of PD and connect adjacent plant cells (Tee and Faulkner, 2024). Theoretically, this endomembrane continuity could provide luminal virions with a potential symplastic route to travel from cell to cell (Goodin et al., 2007). However, plant rhabdovirus particles have never been detected within or surrounding desmotubules. A fundamental unresolved question arises as to whether these endomembrane-trapped virus particles represent terminal assembly products incapable of subsequent cell-to-cell movement or systemic trafficking.

In this study, we aim to revisit this question by integrating ultrastructural characterization and virus reverse genetics analyses. Owing to the distinct particle morphology, our TEM images provide clear evidence that enveloped virions of both RSMV and TYMaV can invade and stably persist within the lumen of mature xylem vessels. Together with the earlier discovery of lettuce necrotic yellows virus particles in the xylem vessels of infected *N. glutinosa* (Chambers and Francki, 1966), these results support that xylem colonization represents a conserved tissue tropism for plant rhabdoviruses. In addition, we identified enveloped virions of both viruses within the phloem SEs, the canonical vascular conduit for systemic viral movement. In line with these ultrastructural findings, analyses of the phenotype of TYMaV-ΔM:RFP further supported the contribution of enveloped virions to vascular viral dissemination.

The detection of enveloped particles within mature VEs appears paradoxical, as these terminally differentiated cells lose their protoplasts and thus lack functional cellular machinery to support viral replication and assembly. This phenomenon can be explained by a temporal window model, which has been previously proposed for non-enveloped viruses (Opalka et al., 1998; Verchot et al., 2001; Wan et al., 2015; Sun et al., 2022; Sun and Folimonova, 2022). The model proposes that viral invasion occurs in immature, physiologically active xylem precursor cells, in which viral replication and assembly occur. Subsequent PCD and cellular autolysis eliminate the protoplast, releasing assembled virions into the hollow lumen of mature xylem vessels. This model is consistent with our observation of TYMaV particles in maturing xylem vessels of infected *N. benthamiana* (Figure 5B). Notably, immature xylem vessels were not identified in the vasculature of infected rice plants; however, xylem parenchyma cells surrounding mature vessels contained highly abundant enveloped particles, reinforcing the strong xylem tropism of RSMV (Figure 2A). Unlike dicots that develop secondary vascular tissues from vascular cambium, monocots such as rice lack bifacial vascular cambium and produce only primary xylem derived from procambial cells (Scarpella and Meijer, 2004). This developmental feature limits the window available for the detection of viral colonization of immature rice xylem.

Although mature phloem SEs are metabolically living cells, they lack most organelles and ribosomes, rendering them likely incompetent to support rhabdovirus replication and morphogenesis. The detection of enveloped rhabdovirus virions within SEs raises an important question of where and when envelopment occurs relative to phloem entry. Two plausible hypotheses may explain this observation. First, enveloped particles may be assembled in CCs and then loaded into SEs through PPUs. A similar trafficking strategy has been proposed for the phloem loading of membrane-associated TuMV VRCs (Wan et al., 2015), which exhibit dimensional and morphological similarities to rhabdovirus enveloped particles. Despite having a larger size exclusion limit than conventional PD (~70 kDa) (Paultre et al., 2016), PPUs still cannot accommodate the passive passage of intact virions or large vesicles. Therefore, viral entry into SEs would require substantial dilation and gating of PPU apertures. Second, rhabdovirus NCs may invade immature SE precursors through ordinary PD prior to SE maturation. These undifferentiated vascular cells retain complete metabolic machinery, enabling viral replication and *de novo* envelopment to generate mature virions observed in conducting cells. SE differentiation is a persistent developmental process in dicots with secondary vascular growth (Hardtke, 2023) and in elongating monocot internodes (Kawahara and Chonan, 1968). Notably, a large number of virions in SEswere observed in bundles enclosed in a vesicle-like membrane (Figures 4B, 5F; Supplementary Figures S5, S8A), supporting *in situ* envelopment in undifferentiated SE precursors rather than virion translocation through PPUs, thereby favoring the second hypothesis.

Integrating our findings with previous data, we propose a model for plant rhabdovirus systemic trafficking, in which enveloped virions may serve as a major mobile entity that might utilize both phloem and xylem vascular conduits for systemic spread (Figure 7). Following initial cell-to-cell movement via MP-gated PD, NCs invade vascular tissues to initiate systemic infection. For xylem-mediated trafficking, NCs colonize metabolically active immature xylem precursor cells from adjacent xylem parenchyma via PD. Viral replication and envelopment occur within these living precursor cells. Upon PCD and cell autolysis, enveloped virions are liberated into the hollow xylem lumen and transported upward along the transpiration stream. For phloem-dependent movement, NCs traffic into CCs to complete full replication and morphogenesis, producing progeny NCs and mature enveloped virions that are subsequently loaded into differentiating or mature SEs through modified PD/PPUs. Viral replication and envelopment could occur in undifferentiated SE precursors. Virions traffic through the phloem along the source-to-sink flow of photoassimilates for long-distance movement. The contribution of enveloped virions to efficient long-distance trafficking may reflect an adaptive strategy to protect viral NCs from degradation by hydrolytic enzymes abundant in vascular sap. Consistently, many positive-sense RNA viruses also rely on intact coat proteins or assembled capsids for stable phloem long-distance movement (Tilsner et al., 2014; Hipper et al., 2013).

**Figure 7:**
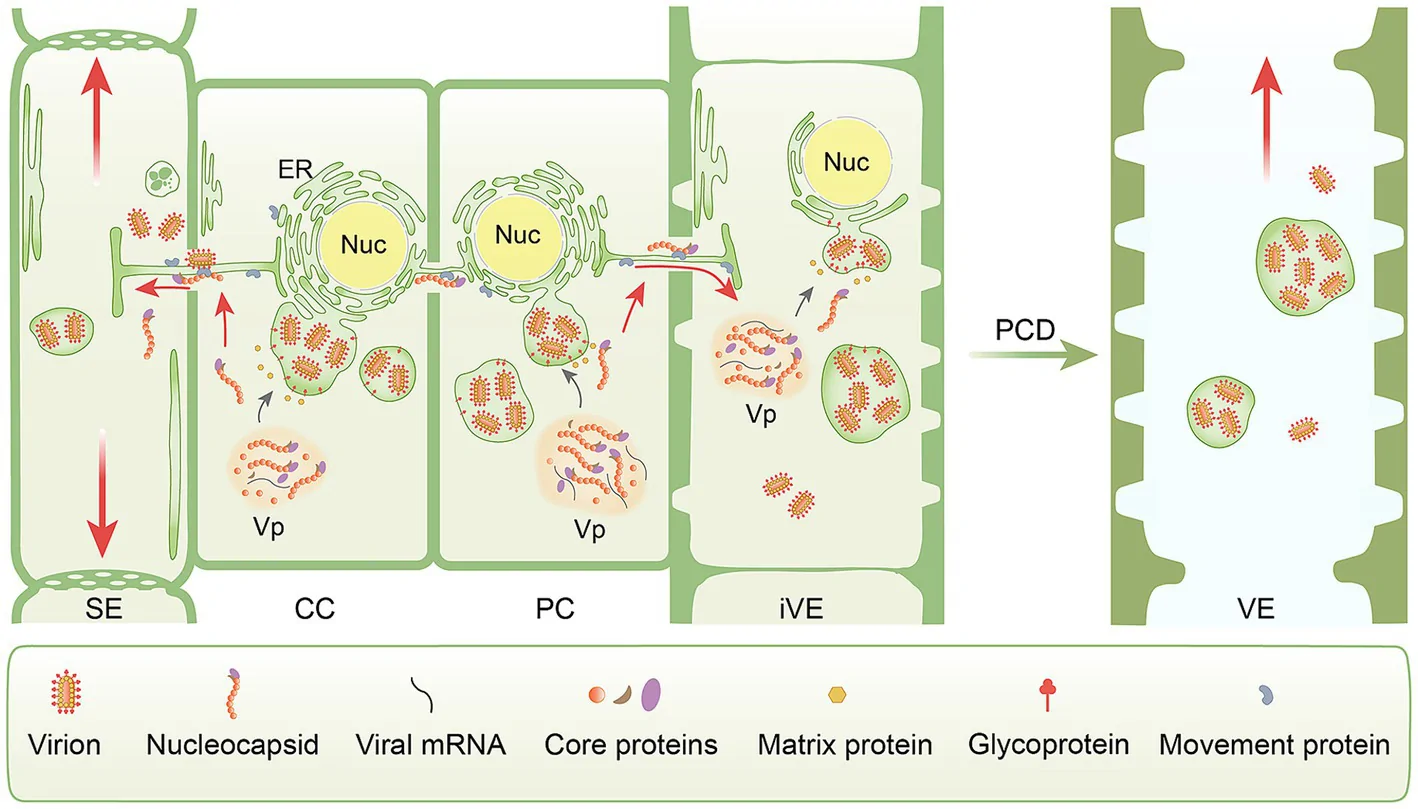
Proposed model for long-distance transport of rhabdoviruses through the xylem and phloem. For xylem-mediated trafficking, viral nucleocapsids (NCs) traffic through plasmodesmata (PD) into immature xylem vessel elements (iVEs), in which viral replication and envelopment occur. iVEs undergo programmed cell death (PCD) and cell autolysis during maturation, and enveloped virions are liberated into the hollow lumen of differentiated vessel elements (VEs) and transported upward along the transpiration stream. For phloem-dependent movement, NCs move into CCs to complete full replication and morphogenesis, producing progeny NCs and mature enveloped virions that are subsequently loaded into differentiating or differentiated sieve elements (SEs) through modified PD or pore-plasmodesma units (PPUs). Viral replication and envelopment may occur in SE precursors. Virions traffic through the phloem along the source-to-sink flow of photoassimilates for bidirectional long-distance movement. ER, endoplasmic reticulum; Nuc, nucleus; Vp, viroplasm.

Notably, our model does not exclude rhabdovirus NCs as an alternative, less-efficient long-distance movement entity. This possibility is supported by reverse-genetic studies of sonchus yellow net virus (SYNV), a nucleorhabdovirus, in which *M* or *G*-gene deletion mutants retained limited systemic infectivity, albeit with drastically reduced infection efficiency (approximately 9 and 14% of wild-type levels), delayed symptom development, and a vasculature-restricted tropism (Wang et al., 2015; Ma and Li, 2020). In addition, multiple naturally occurring plant rhabdovirus species/isolates with truncated or deficient M and/or G genes have recently been identified through high-throughput sequencing or metatranscriptomic data mining, although the infectivity of these putative viruses remains to be experimentally validated (Zhang et al., 2021; Bejerman et al., 2021; Lee et al., 2022; Kauffmann et al., 2022; Bejerman et al., 2023; Botermans et al., 2026). Nevertheless, these data suggest that at least for some rhabdoviruses, their NCs alone are capable of inefficient systemic trafficking. We also cannot exclude the possibility that the M protein may facilitate systemic movement through functions independent of virion envelopment, such as the suppression of host hormone-mediated antiviral defenses (Wu et al., 2026b). However, the systemic movement defect of TYMaV-ΔM:RFP, together with the detection of enveloped virions in vascular conduits, supports an important role for mature virions in efficient long-distance movement, while the contribution of M to virion morphogenesis cannot yet be fully separated from other functions of M.

Beyond functioning in intra-plant systemic trafficking, vascular-localized enveloped virions also bear important implications for inter-plant viral transmission. Enveloped plant rhabdoviruses are exclusively transmitted by sap-feeding hemipteran insects (e.g., aphids, leafhoppers, planthoppers, and whiteflies) in a circulative-propagative manner, in which viruses replicate and disseminate from midgut to salivary glands before transmission to new host plants (Dietzgen et al., 2020). Successful insect acquisition requires intact virions, as the envelope-resident surface G protein mediates receptor recognition and endocytic entry into insect midgut epithelial cells (Whitfield et al., 2018). Given that hemipteran vectors feed on both phloem and xylem sap (Lei et al., 2001; Seo et al., 2009; Pompon et al., 2011), the presence of intact virions in both vascular conduits provides dual sources for viral acquisition. Future investigations are warranted to dissect the distinct and relative contributions of the phloem versus xylem-resident virions to viral systemic trafficking and insect-mediated horizontal transmission.

## Data Availability

The original contributions presented in the study are included in the article/Supplementary material, further inquiries can be directed to the corresponding authors.
