# Supplementary material for: Potential contribution of enveloped virions to long-distance movement of plant rhabdoviruses
Source: Front Microbiol. 2026 Oct 19;17:1994490. doi: 10.3389/fmicb.2026.1994490 (PMC13724602; doi:10.3389/fmicb.2026.1994490)
Supplement: Supplementary file 1 [file Data_Sheet_1.pdf]

Supplementary Material

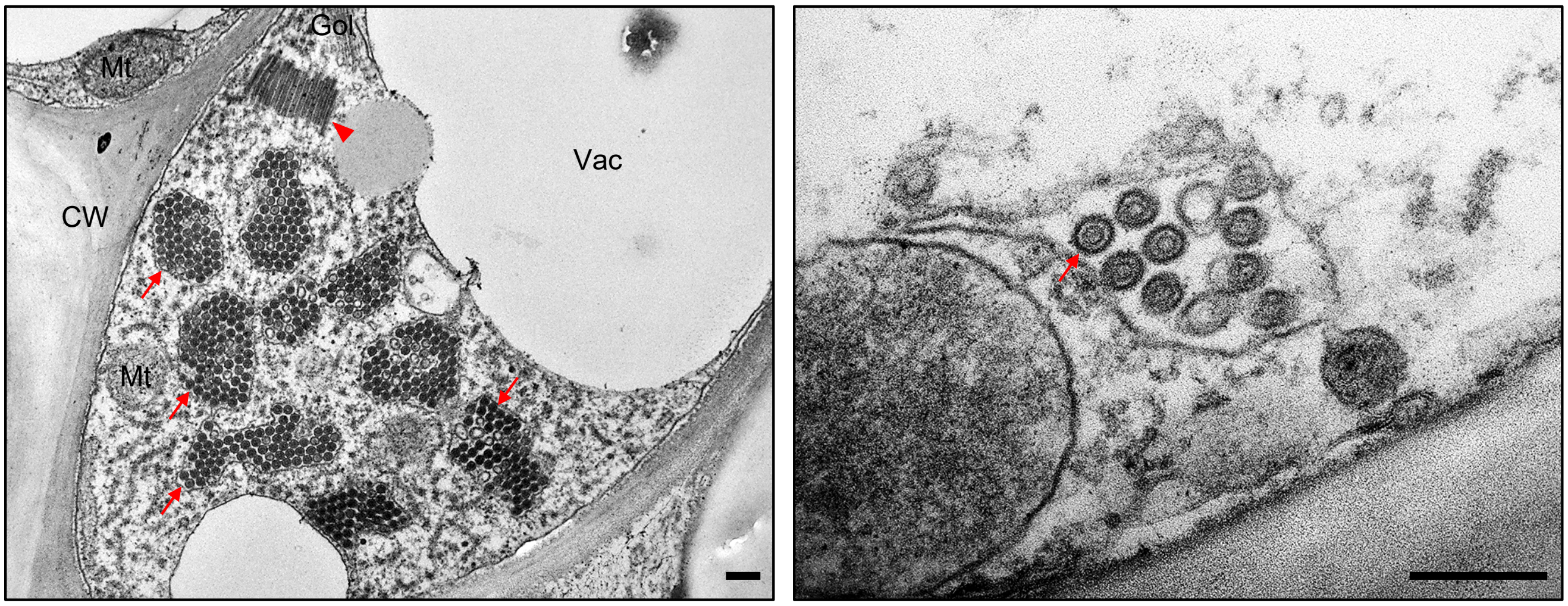

Supplementary Figure S1. Representative TEM images of RSMV particles in transverse sections of infected rice leaf tissues. Arrows indicate enveloped virions, which frequently occur in clusters surrounded by vesicle-like membranes, whereas arrowheads indicate non-enveloped cores. CW, cell wall; Gol, Golgi apparatus; Mt, mitochondrion; Vac, vacuole. Scale bars, 200 nm.

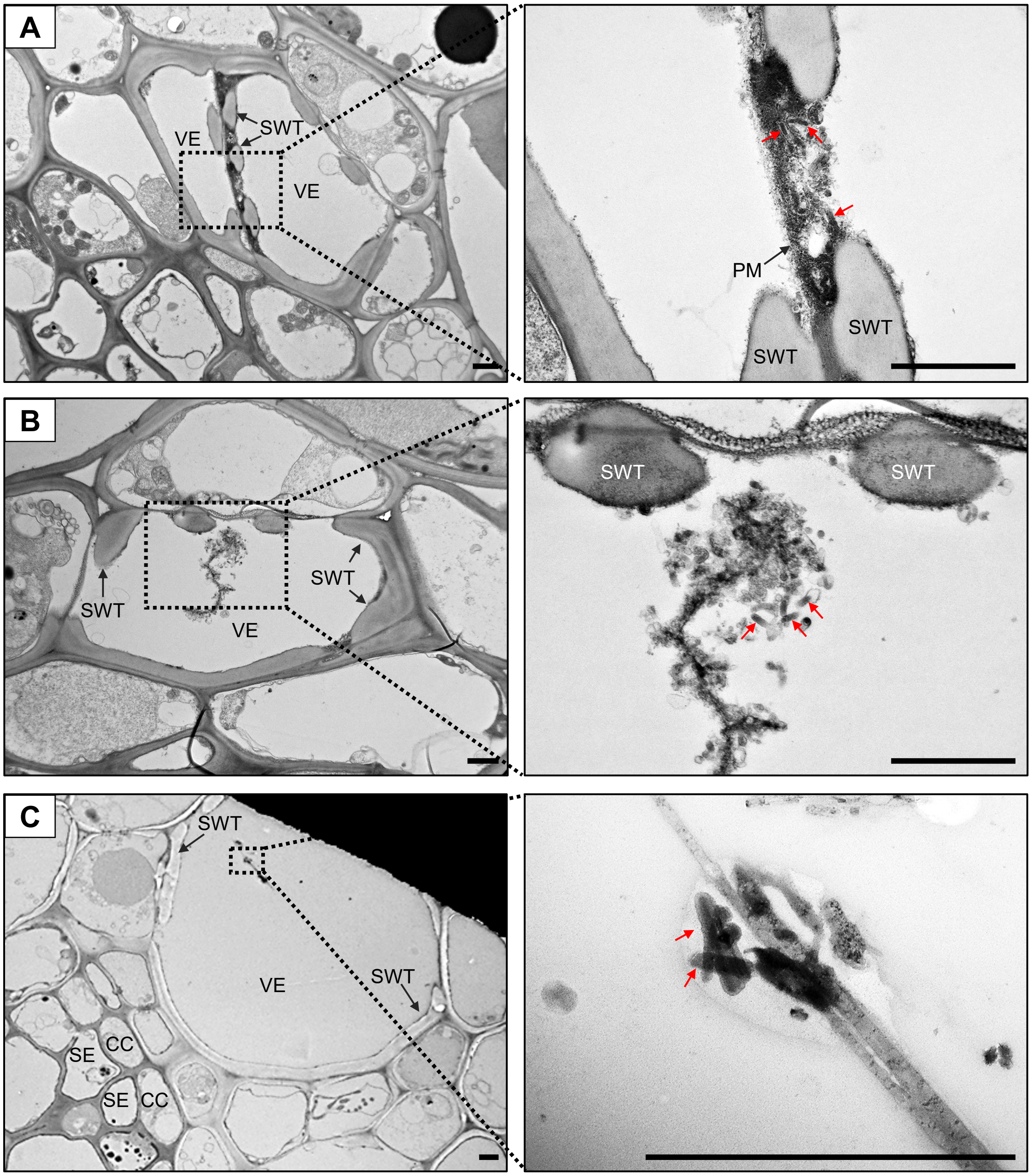

Supplementary Figure S2. TEM observation of RSMV particles in vessel elements of rice. (A, B) Enveloped virus particles in vessel elements of rice leaf samples. (C) Enveloped virus particles in a vessel element of a rice stem sample. Red arrows indicate virus particles. VE, vessel element; SWT, secondary cell wall thickening; PM, pit membrane; SE, sieve element; CC, companion cell. Scale bars, 1 μm.

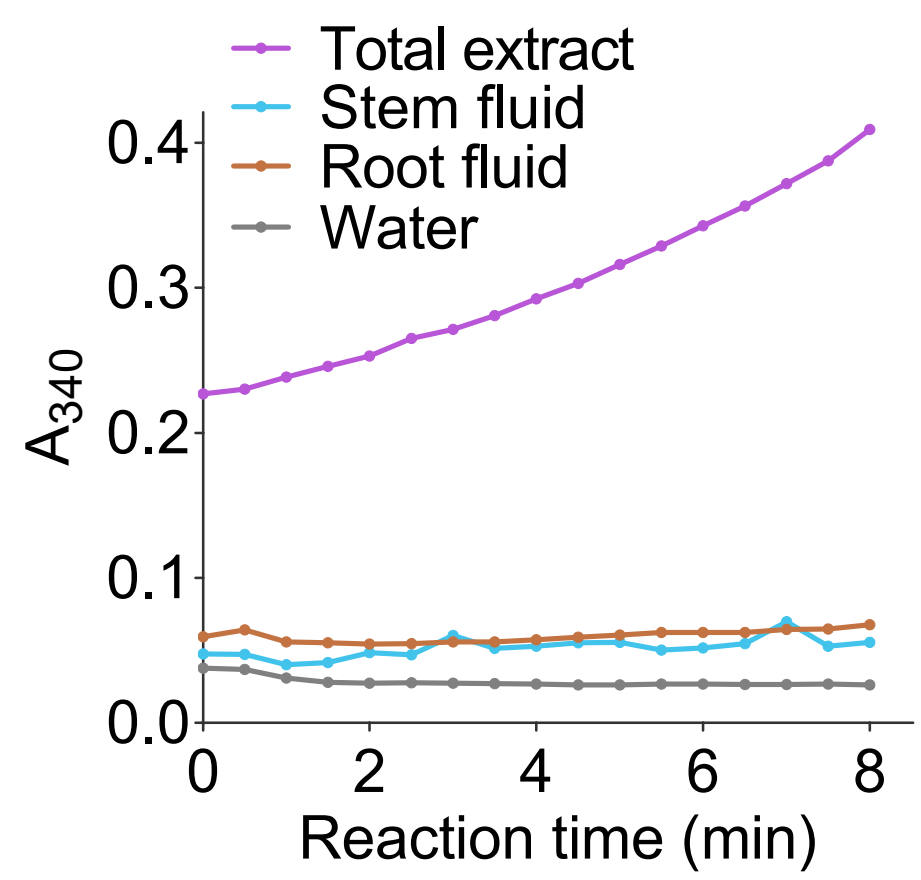

Supplementary Figure S3. Analysis of cytoplasmic contamination in the xylem sap samples collected from RSMV-infected rice plants via G6PDH activity assay. The increase in absorbance at 340 nm (A_{340}) resulting from NADPH production was monitored over 8 min at a 2-min interval. Total protein extracts from rice tissues and deionized water were used as positive and blank controls, respectively.

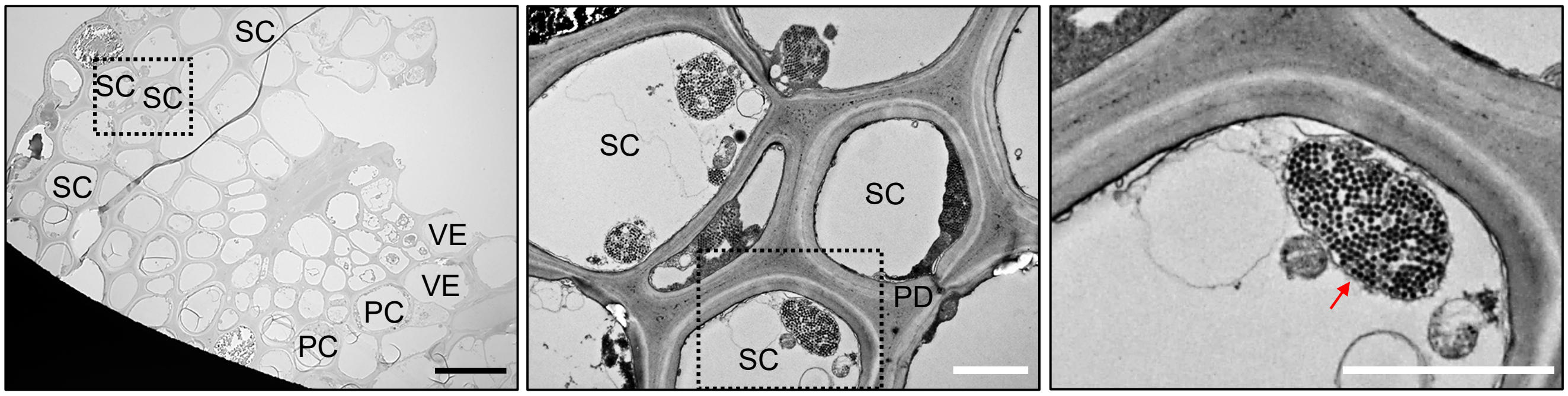

Supplementary Figure S4. TEM images showing the accumulation of enveloped RSMV virions in sclerenchyma cells (SCs) surrounding the vascular bundles in infected rice leaves. SCs have uniformly thickened secondary cell walls and nearly complete degradation of cellular contents. Enveloped virions occurred in clusters surrounded by vesicle-like membranes. Regions shown at successive higher magnifications are indicated by black dashed boxes, and arrows indicate virus particles. PD, plasmodesma; VE, vessel element; PC, parenchyma cell. Black and white scale bars indicate 10 μm and 2 μm, respectively.

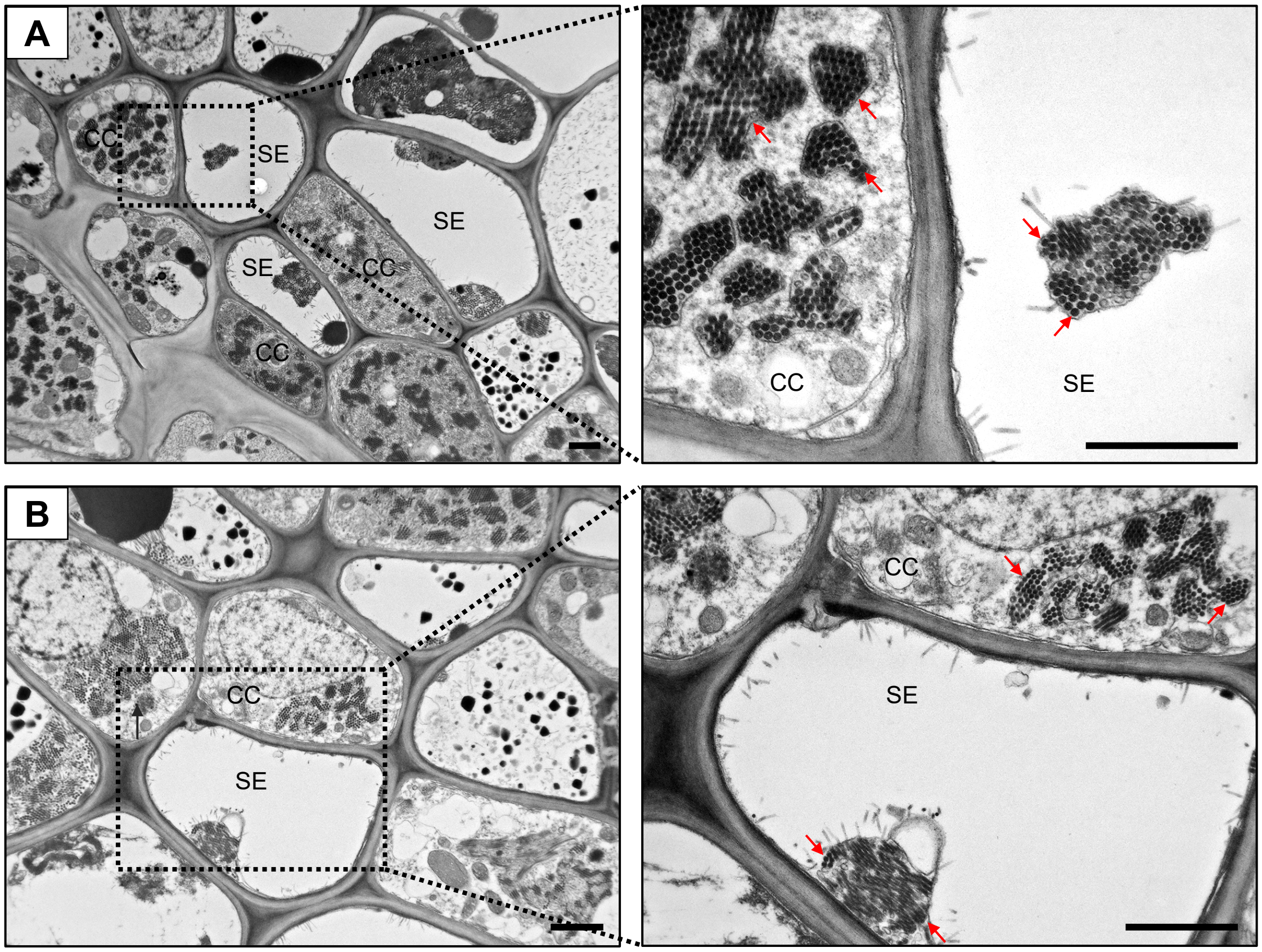

Supplementary Figure S5. TEM observation of RSMV particles in sieve element–companion cell complexes of rice. (A, B) Two different fields of view showing enveloped virus particles in both sieve elements and companion cells, often enclosed within vesicles. Red arrows indicate virus particles. SE, sieve element; CC, companion cell. Scale bars, 1 μm.

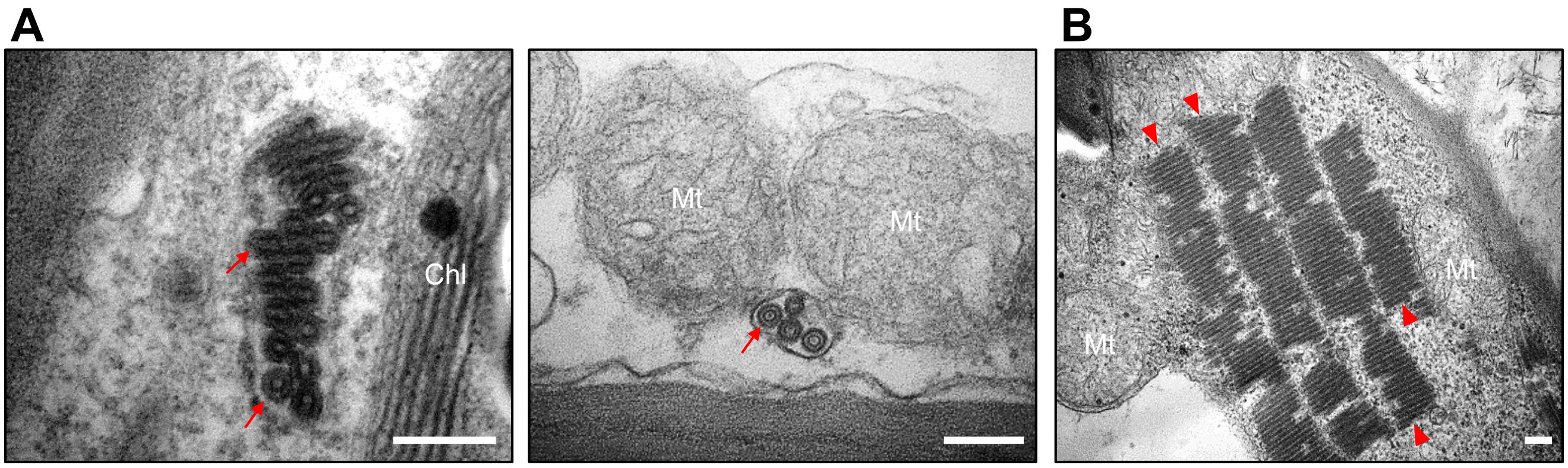

Supplementary Figure S6. Representative TEM images of TYMaV particles in transverse sections of infected Nicotiana benthamiana leaf mesophyll cells. (A) Enveloped virions (arrows) occur in clusters surrounded by vesicle-like membranes. (B) Non-enveloped cores (arrow heads) lacking an envelope and enclosing membrane occur in orderly arrays. Chl, chloroplast; Mt, mitochondrion. Scale bars, 200 nm.

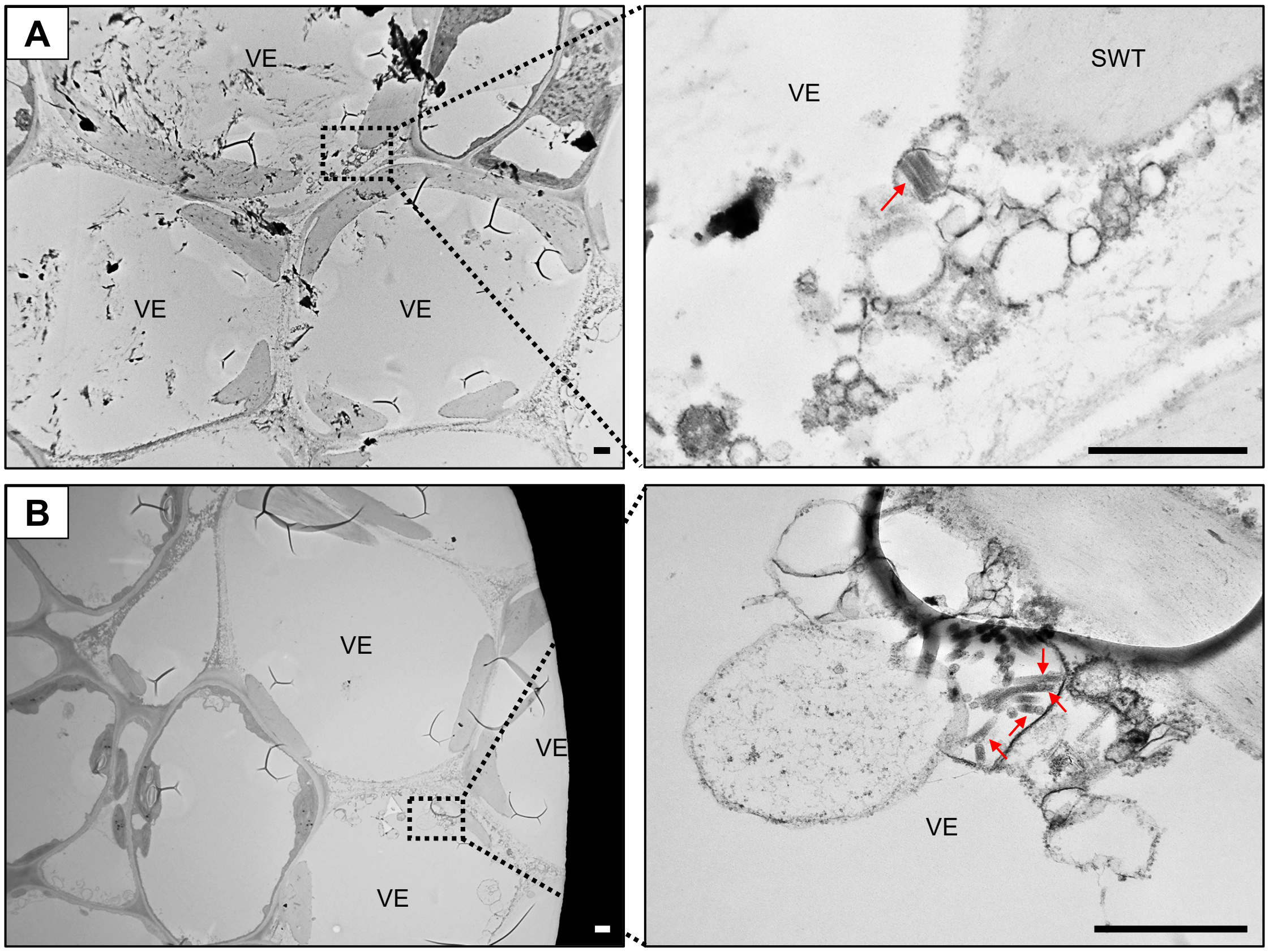

Supplementary Figure S7. TEM observation of TYMaV particles in vessel elements of *Nicotiana benthamiana* leaves. (A, B) Two different fields of view showing enveloped virus particles enclosed within vesicles. Red arrows indicate virus particles. VE, vessel element; SWT, secondary cell wall thickening. Scale bars, 1 µm.

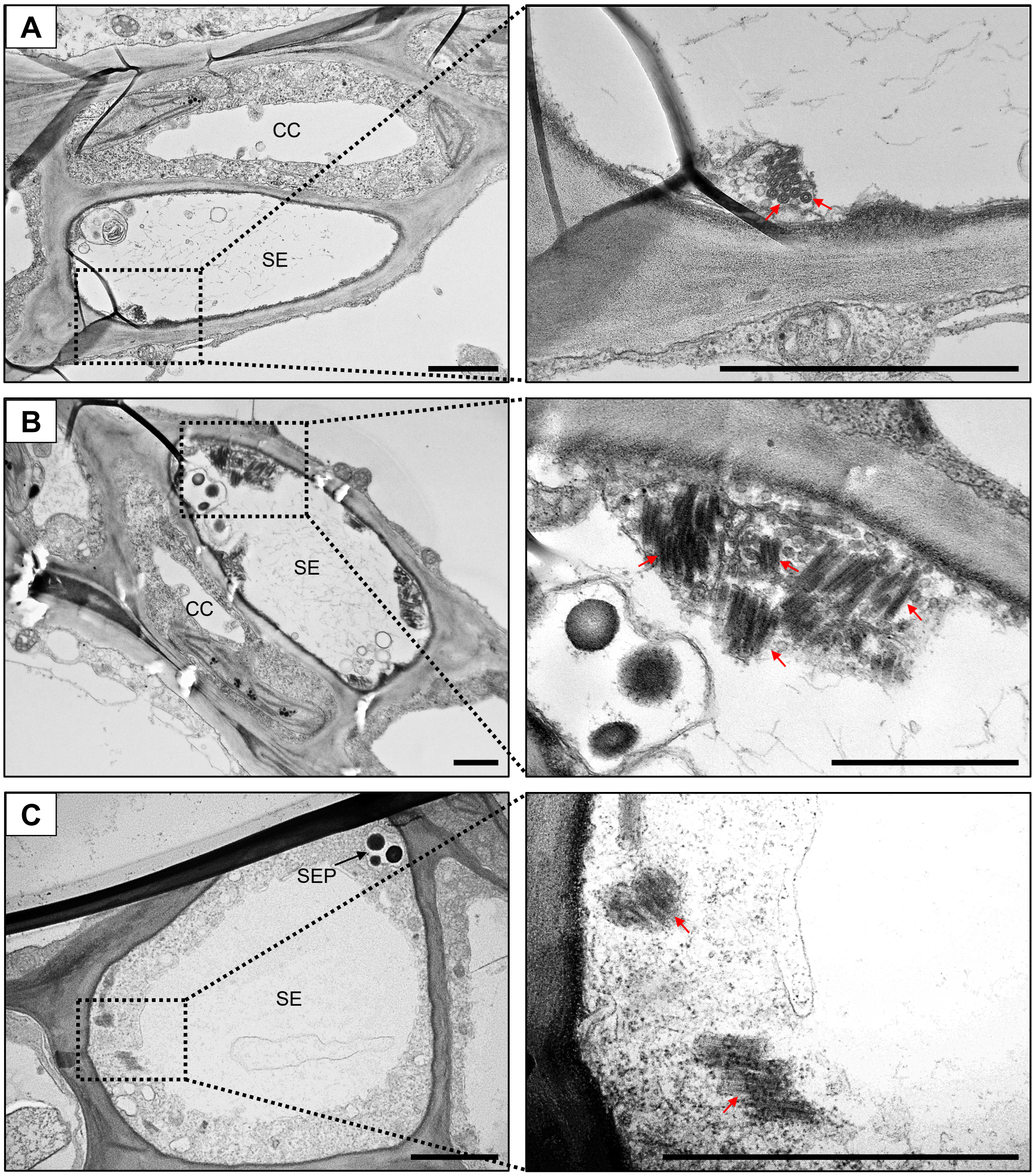

Supplementary Figure S8. TEM observation of TYMaV particles in sieve elements of *Nicotiana benthamiana*. (A–C) Three different fields of view showing enveloped virus particles in sieve elements. Red arrows indicate virus particles. SE, sieve element; CC, companion cell; SEP, sieve element plastid. Scale bars, 1 μm.
